# The Ratio of Treg/Th17 Cells Correlates with the Disease Activity of Primary Immune Thrombocytopenia

**DOI:** 10.1371/journal.pone.0050909

**Published:** 2012-12-03

**Authors:** Lili Ji, Yanxia Zhan, Fanli Hua, Feng Li, Shanhua Zou, Weiguang Wang, Dongli Song, Zhihui Min, Hao Chen, Yunfeng Cheng

**Affiliations:** 1 Department of Hematology, Zhongshan Hospital, Fudan University, Shanghai, China; 2 Biomedical Research Center, Zhongshan Hospital, Fudan University, Shanghai, China; 3 Department of Surgery, Huadong Hospital, Fudan University, Shanghai, China; 4 Department of Hematology, Jinshan Hospital, Fudan University, Shanghai, China; New York University, United States of America

## Abstract

**Background:**

Primary immune thrombocytopenia (ITP) is an autoimmune heterogeneous disorder that is characterized by decreased platelet count. Regulatory T (Treg) cells and T helper type 17 (Th17) cells are two subtypes of CD4^+^ T helper (Th) cells. They play opposite roles in immune tolerance and autoimmune diseases, while they share a common differentiation pathway. The imbalance of Treg/Th17 has been demonstrated in several autoimmune diseases. In this study, we aimed to investigate the ratio of the number of Treg cells to the number of Th17 cells in ITP patients and evaluate the clinical implications of the alterations in this ratio.

**Methods:**

Thirty adult patients with newly diagnosed ITP enrolled in this study. Twelve patients had been clinically followed up for 12 months. The percentages of CD4^+^CD25^hi^Foxp3^+^ Treg cells and CD3^+^CD4^+^IL-17-producing Th17 cells in these patients and healthy controls (n = 17) were longitudinally analyzed by flow cytometry.

**Results:**

The percentage of Treg cells in ITP patients was significantly lower than that of healthy controls, and the percentage of Th17 cells increased significantly at disease onset. The ratio of Treg/Th17 correlated with the disease activity.

**Conclusion:**

The ratio of Treg/Th17 might be relevant to the clinical diversity of ITP patients, and this Treg/Th17 ratio might have prognostic role in ITP patients.

## Introduction

Primary immune thrombocytopenia (ITP) is an immune-mediated heterogeneous disorder characterized by decreased platelet count and increased risk of bleeding. Patients present different severities of thrombocytopenia and different responses to corticosteroids. Some patients have either no symptoms or minimal bruising, while other patients are at a risk of serious bleeding, which may include fatal intracranial hemorrhage, gastrointestinal hemorrhage, extensive skin and mucosal hemorrhage. The severity of thrombocytopenia correlates to some extent with the bleeding risk. Concepts surrounding the mechanisms of thrombocytopenia in ITP have shifted from the traditional view of increased platelet destruction mediated by autoantibodies to mechanisms in which both impaired platelet production and T cell-mediated effects play a role [1]–[3].

Semple *et al* [4] first reported the T cell had reactivity against platelets, which initiated the investigation on T cell disorder in ITP. CD4^+^ cells can orchestrate host responses through the release of distinct cytokine profiles. CD4^+^CD25^high^Foxp3^+^Treg and CD3^+^CD4^+^IL-17-producing Th17 are two subsets of CD4^+^ Th cells. Autoreactive CD4^+^ Th cells against platelet GPIIb-IIIa antigen were identified and demonstrated to help B cells produce autoantibodies [5], [6]. CD4^+^Th cell defects were then recognized as an important part in the pathogenic process of ITP. Decreased number and function of Treg cells has been demonstrated in ITP patients [7]. While Treg cells play a fundamental role in the maintenance of immune tolerance to prevent autoimmune disease, Th17 cells play the opposite role. They secrete IL-17 and other pro-inflammatory cytokines, leading to consequent inflammation cytokines recruitment and tissue injury [8]. Th17 cells are thought to be involved in inflammatory and autoimmune disease [9], [10]. Some researchers argued that Th17 cells count did not differ between ITP patients and normal controls [11], [12]. However, other groups reported up-regulation of Th17 cells in ITP patients [13]–[16]. So far, the role of Th17 cells in ITP is still in contention.

Recent studies show that there is a dichotomy in the generation of Treg cells and Th17 cells [17], [18]. The TGF-β signaling causes the common precursors to pass through an intermediate stage characterized by the co-expression of Foxp3 and RORγ-t [19]. TGF-β alone favors Foxp3 expression and paradoxically inhibits RORγ-t transcriptional activity, while it drives Th17 differentiation in combination with IL-1β and IL-23 or IL-21, IL-6 in human [20]. Some studies showed that human peripheral blood and lymphoid tissue contain a significant number of Treg cells that have the capacity to produce IL-17 upon activation, which indicating the inner connection of these two T cell subtypes [21]–[23]. Thus, the Treg/Th17 balance is regarded as a key factor in immune homeostasis. Treg/Th17 imbalance has been found associated with disease activity in several autoimmune diseases [24]–[27]. This made us interested in investigating the Treg/Th17 imbalance profile among ITP patients of different disease states and normal volunteers, and in finding out whether the imbalance associate with the clinical characteristics of ITP. This investigation into the immune disorder of ITP may help improve clinical management and therapeutic options.

## Materials and Methods

### Patients

Thirty adult patients with newly diagnosed ITP according to the ITP diagnosis criteria proposed by an international working group [28], [29], were enrolled in this study (21 females and 9 males, age range 21–80 years, median 52 years, Table 1). Among them, 12 patients were asymptomatic and their platelet count remained over 30×10^9^/L stably. These patients were given no treatment other than observation (observation group, OB group). The other 18 patients were newly diagnosed and required treatment (requiring treatment group, RT group) due to clinically significant bleeding and/or extremely low platelet count. Seventeen healthy volunteers were taken as normal controls (normal control group, NC group). The RT group patients received first-line corticosteroids treatment according to international consensus [28]–[30]. Secondary ITP, pregnant patients and those who were unable to undergo glucocorticoid therapy were excluded. When enrolled, 2 ml venous blood samples of patients and normal controls were collected. One month after the initial treatment, 14 RT patients’ efficacy was validated in accordance with the Vicenza Consensus Conference [28] and 2 ml venous blood samples of the patients (n = 14) were collected. Among them, 8 patients were also assessed at 2 months and 12 months after the treatment, when 2 ml venous blood collection were performed (n = 8). Four OB patients were followed up and had 2 ml venous blood samples collected at 1 month, 2 months and 12 months after the enrollment. The clinical characteristics of these subjects are listed in Table 1.

**Table 1:** Clinical characteristics of ITP patients.

Patient No.	Sex	Age	Platelet counts(×10^9^/L)	
		(years)	Pre-treatment	Post-treatment
RT1	M	69	2	104
RT2	M	27	21	146
RT3	F	33	2	217
RT4	F	35	22	207
RT5	F	51	4	9
RT6	F	24	18	172
RT7	F	76	15	–
RT8	F	21	7	245
RT9	M	59	17	23
RT10	M	69	2	2
RT11	F	52	9	–
RT12	F	55	6	–
RT13	M	24	29	10
RT14	F	56	24	125
RT15	F	73	18	214
RT16	F	43	20	161
RT17	M	29	4	–
RT18	F	47	16	151
OB1	F	46	33	–
OB2	F	57	33	–
OB3	F	68	32	–
OB4	F	49	57	–
OB5	F	52	41	–
OB6	F	58	30	–
OB7	F	61	30	–
OB8	M	77	22	–
OB9	F	80	37	–
OB10	M	31	41	–
OB11	M	36	38	–
OB12	F	70	42	–
	M:F = 9∶21	52(21–80)	22(2–57)	149(2–245)

RT: ITP patients requiring treatment; OB: observation ITP patients;

The study was approved by the respective local Medical Ethics Committees of Zhongshan Hospital of Fudan University. Written informed consent was obtained from each patient before being included in the study.

### Samples Preparation

Venous blood samples were collected in 2-mL ethylenediaminetetraacetic acid-treated tubes and diluted 1∶2 with Hanks balanced salt solution (HBSS) before Ficoll-Hypaque gradient centrifugation (2,200 rpm at room temperature for 15 min). Washed and resuspended, isolated peripheral blood mononuclear cells (PBMCs) were cryopreserved in fetal bovine serum containing 10% dimethyl sufloxide (DMSO), and stored in liquid nitrogen for future flow cytometric analysis (FCM).

### Flow Cytometric Analysis

Cryopreserved PBMCs were thawed at 37°C, washed twice with HBSS, and stained with trypan blue to test cell viability. 1×10^6^ PBMC were distributed for Treg Flow Cytometric analysis. Cells were stained with CD4 PE-Cy5, CD25 PE, and Foxp3 Alexa Flour®488 antigens according to the manufacturer’s protocol (Human Treg Flow™ Kit, Biolegend, 320401). To detect Th17 cells, PBMCs were adjusted concentration as 5×10^5^/ml in RPMI1640 medium supplemented with 10% heat-inactivated fetal bovine serum, 2 mM L-glutamine, 200 U/ml penicillin, and 100 µg/ml streptomycin. The PBMCs were incubated for 4 hours with 50 ng/ml phorbol myristate acetate (PMA, sigma-aldrich, P8139) and 500 ng/ml ionomycin (sigma-aldrich, I9657). In the later 2 hours, 1 ul/ml brefeldin A solution (BFA, Biolegend, 420602) was added into the culture system. Then PBMCs were stained with CD3 FITC, CD4 PE, IL-17 Alexa Flour®647 antigens according to the manufacturer’s instructions (Human Th17 Flow™ Kit, Biolegend, 339401). Stained cells were tested on a FACS Aria II flow cytometer (BD, USA) and then analyzed using Flowjo software version 7.6.1.

### Statistical Methods

All analyses were performed with STATA 7.0 software. Data were expressed as mean ± SD. Normality was assessed by Shapiro-Wilk W test. In pairwise comparison, student *t* test and Wilcoxon rank-sum (Mann-Whitney) test were used for data fulfilled normal distribution and for those did not, respectively. When multiple groups were compared, One Way ANOVA and Kruskal Wallis test were used for data fulfilled normal distribution and for those did not, respectively. For all tests, two-sided *p* values less than 0.05 were considered statistically significant.

## Results

### Treg/Th17 Balance of ITP Patients Skewed Toward Th17

**Figure 1:**
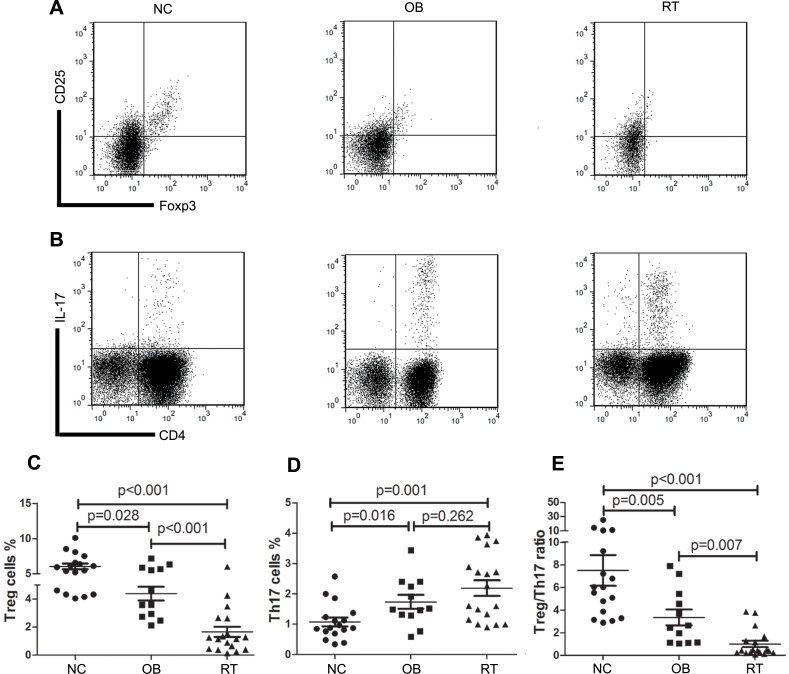
Treg/Th17 balance of ITP patients skewed toward Th17. A. Representative dot plots of Treg cells (CD4^+^CD25^hi^Foxp3^+^ cells) in NC, OB and RT group. B. Representative dot plots of Th17 cells (CD3^+^CD4^+^IL-17^+^ cells) in NC, OB and RT group. C. The mean ± SD of the percentage of Treg cells in different group. D. The mean ± SD of the percentage of Th17 cells in different group. E. The mean ± SD of the Treg/Th17 ratio in different group. *p* value was shown in the figure. NC: normal control; OB: observation ITP patients; RT: ITP patients requiring treatment.

The 3 groups (NC group, OB group and RT group) were age and sex matched (Table 2). The percentage of Treg cells in PBMCs of ITP patients and normal controls were determined by FCM (Fig. 1A). The results showed a notable decrease of Treg cells in both ITP groups compared with NC group (NC vs OB: (6.04±1.70)% vs (4.39±1.70)%, p = 0.028; NC vs RT: (6.04±1.70)% vs (1.66±1.58)%, p<0.001; Fig. 1C). OB group, as the relatively moderate disease, possessed significant higher Treg cells percentage than RT group did (p<0.001; Fig. 1C).

A population of CD3^+^CD4^+^IL-17^+^ cells was identified as Th17 cells in either ITP group (Fig. 1B), which was significantly larger than that in normal controls (NC vs OB: (1.08±0.59)% vs (1.74±0.79)%, p = 0.016; NC vs RT: (1.08±0.59)% vs (2.19±1.10)%, p = 0.001; Fig. 1D). There was no significant Th17 cells percentage difference between the OB and RT groups (p = 0.262; Fig. 1D).

As a result of the alteration of both cell subtypes, the ratio of Treg cells to Th17 cells dropped significantly (NC vs OB: (7.50±5.53) vs (3.36±2.43), p = 0.005; NC vs RT: (7.50±5.53) vs (1.01±1.21), p<0.001; Fig. 1E). The Treg/Th17 ratio of OB group was higher than that of RT group (p = 0.007).

**Table 2:** Comparisons of multiple parameters in different groups.

	NC	OB	RT	p value
sex(M:F)	7∶10	3∶9	6∶12	0.667
age(median,y)	45	57.5	49	0.081
platelet count(median,×10^9^/L)	201	35	15.5	<0.001
Treg(mean ± SD,%)	6.04±1.70	4.39±1.70	1.66±1.58	<0.001
Th17(mean ± SD,%)	1.08±0.59	1.74±0.79	2.19±1.10	0.002
Treg/Th17 ratio (mean ± SD)	7.50±5.53	3.36±2.43	1.01±1.21	<0.001

RT: ITP patients requiring treatment; OB: observation ITP patients;

### Treg/Th17 Balance Deviation was Associated with Disease Activity

**Figure 2:**
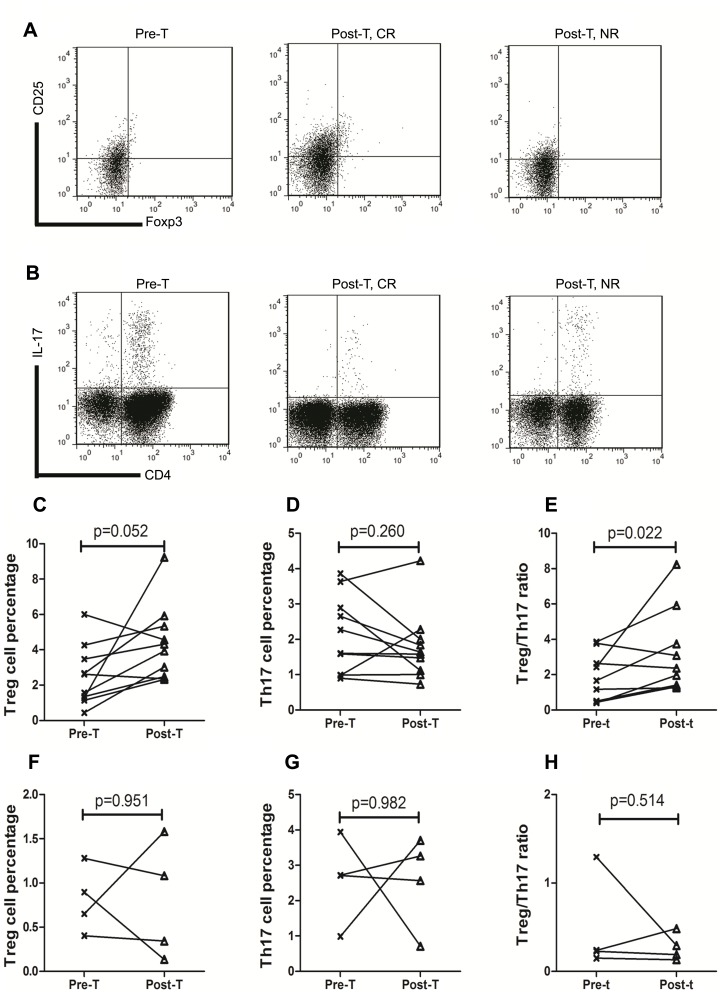
Treg/Th17 ratio deviated along with disease activity. A. Representative dot plots of Treg cells (CD4^+^CD25^hi^Foxp3^+^ cells) in Pre-T, Post-T CR and Post-T NR group. B. Representative dot plots of Th17 cells (CD3^+^CD4^+^IL-17^+^ cells) in Pre-T, Post-T CR and Post-T NR group. C. The change of Treg cells percentage in CR patients before and after corticosteroids treatment. D. The change of Th17 cells percentage in CR patients before and after corticosteroids treatment. E. The change of Treg/Th17 ratio in CR patients before and after corticosteroids treatment. F. The change of Treg cells percentage in NR patients before and after corticosteroids treatment. G. The change of Th17 cells percentage in NR patients before and after corticosteroids treatment. H. The Treg/Th17 ratio of before and after corticosteroids treatment in NR patients. *p* value was shown in the figure. Pre-T: pre-treament; Post-T: post-treatment; CR: complete response; NR: no response.

In RT group, 10 patients attained complete response (CR) with a median platelet count of 166×10^9^/L (range 104–245×10^9^/L), while 4 patients got no response (NR, median platelet count 10×10^9^/L, range 2–23×10^9^/L) at 1 month after corticosteroids therapy. Representative Treg and Th17 FCM pictures of pre-treatment (Pre-T) and post-treatment (Post-T, including CR and NR) patients were showed in Fig. 2A and Fig. 2B.

In CR patients (n = 10), Treg cells percentage increased from (3.32±2.77)% for Pre-T to (4.35±2.13)% for Post-T (p = 0.052, Fig. 2C). Although Th17 cells percentage fell from (2.25±1.23)% (Pre-T) to (1.79±0.97)% (Post-T), there is no statistic difference found (p = 0.26, Fig. 2D). The Treg/Th17 ratio was significantly elevated after remission (Pre-T (1.74±1.29), Post-T (3.07±2.21), p = 0.022; Fig. 2E).

As for the NR patients (n = 4), there was no difference of Treg cells percentage between Pre-T and Post-T (Pre-T (0.81±0.37)%, Post-T (0.78±0.67)%, p = 0.951; Fig. 2F). The percentage of Th17 cells remained almost the same after the treatment (Pre-T (2.59±1.21)%, Post-T (2.56±1.32)%, p = 0.982; Fig. 2G). Thus the Treg/Th17 ratio made no difference between before and after corticosteroids treatment in NR patients (Pre-T (0.48±0.55), Post-T (0.28±0.15), p = 0.514; Fig. 2H).

### Treg/Th17 Imbalance Correlated with Disease Activity in the Long Term

**Figure 3:**
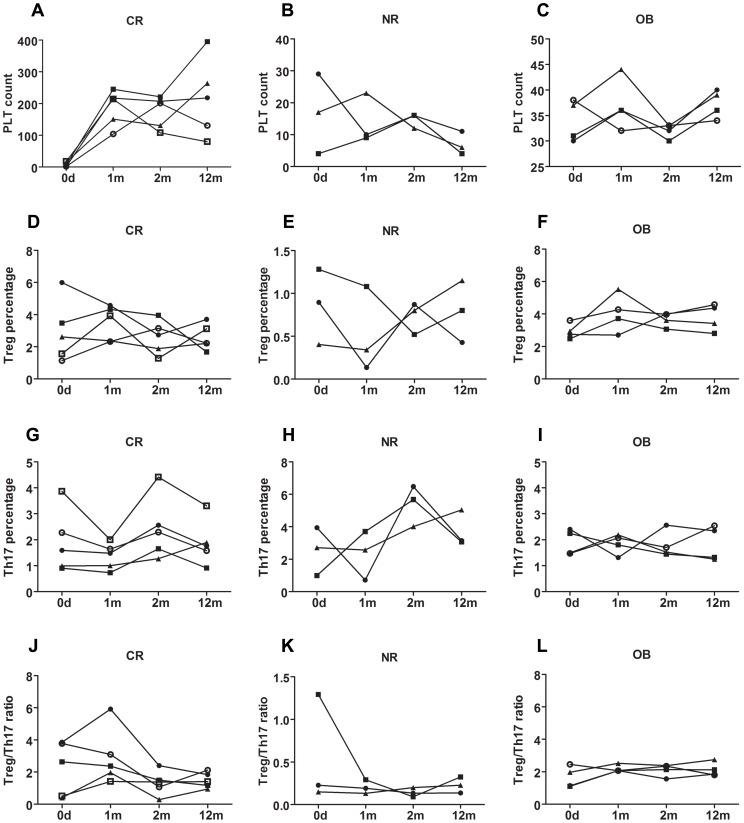
Long term follow-up of platelet count, Treg cells percentage, Th17 cells percentage and Treg/Th17 ratio. A. Platelet count of CR patients at diagnosis, 1month after the initial of treatment, 2 months and 12 months after diagnosis. B. Platelet count of NR patients at diagnosis, 1month after the initial of treatment, 2 months and 12 months after diagnosis. C. Platelet count of OB patients at diagnosis, 1month, 2 months and 12 months after enrollment. D. Treg cells percentage of CR patients at diagnosis, 1month after the initial of treatment, 2 months and 12 months after diagnosis. E. Treg cells percentage of NR patients at diagnosis, 1month after the initial of treatment, 2 months and 12 months after diagnosis. F. Treg cells percentage of OB patients at diagnosis, 1month, 2 months and 12 months after enrollment. G. Th17 cells percentage of CR patients at diagnosis, 1month after the initial of treatment, 2 months and 12 months after diagnosis. H. Th17 cells percentage of NR patients at diagnosis, 1month after the initial of treatment, 2 months and 12 months after diagnosis. I. Th17 cells percentage of OB patients at diagnosis, 1month, 2 months and 12 months after the enrollment. J. Treg/Th17 ratio of CR patients at diagnosis, 1month after the initial of treatment, 2 months and 12 months after diagnosis. K. Treg/Th17 ratio of NR patients at diagnosis, 1month after the initial of treatment, 2 months and 12 months after diagnosis. L. Treg/Th17 ratio of OB patients at diagnosis, 1month, 2 months and 12 months after enrollment. CR: complete response; NR: no response; OB, observation group.

We further followed up 8 treated patients in the long term, whose clinical parameters were detailed in Table 3. During the 1-year follow-up, 5 patients remained remission (4 patients with platelet count over 100×10^9^/L, 1 patient with platelet count of 80×10^9^/L) at 12 months after enrollment (Fig. 3A) and 3 patients with platelet counts kept below 30×10^9^/L (Fig. 3B). Four OB patients were also followed up until 1 year after enrollment, whose platelet count remained over 30×10^9^/L stably (Fig. 3C).

**Table 3:** Clinical parameters of Long-term follow-up patients.

Patient	Disease	WBC(×10^9^/L)				lymphocyte(%)				Platelet counts (×10^9^/L)			
No.	State	0 M	1 M	2 M	12 M	0 M	1 M	2 M	12 M	0 M	1 M	2 M	12 M
RT1	CR	20.73	16.89	5.69	7.02	7.4	6.5	20.6	45	2	104	201	131
RT3	CR	8.43	12.19	15.7	12.02	18.3	24.7	16.9	18.6	2	217	207	218
RT5	NR	12.73	12.43	9.64	12.77	5.7	9.4	4.5	9.6	4	9	16	4
RT8	CR	12.77	14.31	13.57	18.3	18	15.4	16.1	12	7	245	221	395
RT9	NR	11.24	11.2	11.2	7.73	10	7	7	10	17	23	12	6
RT13	NR	13.28	9.92	11.04	8.76	10	14.4	12.8	28	29	10	16	11
RT15	CR	8.94	9.49	11.29	9.38	26.6	23.3	15.3	34.5	18	214	108	80
RT18	CR	8.88	8.4	15.59	8.72	16	8.4	13.5	28.2	16	151	131	264

RT: ITP patients requiring treatment; OB: observation ITP patients;

Two months after the enrollment (ITP patients were enrolled at diagnosis), the Treg cells percentage was significantly higher in CR patients than that in NR patients ((2.60±1.05)% *vs* (0.73±0.19)%, p = 0.025; Fig. 3D, 3E), while the Treg cell percentage of 4 OB patients’ ((3.66±0.44)%, Fig. 3F) was not statistically different from the CR patients’ (p = 0.086). There was no statistic difference among the 3 groups (CR (2.43±1.22)%, NR (5.38±1.25)%, OB (1.81±0.51)%, p = 0.064; Fig. 3G, 3H, 3I) when Th17 cells percentages were compared. The Treg/Th17 cells ratio of CR patients was higher than that of NR patients (CR (1.32±0.76), NR (0.14±0.05), p = 0.025; Fig. 3J, 3K), but no statistic difference was found when compared to OB group ((2.10±0.37), p = 0.142; Fig. 3L).

At 12 months after enrollment, the Treg cells percentages of CR patients ((2.59±0.81)%, p = 0.025) and OB patients ((3.79±0.83)%, p = 0.034) were higher than that of NR patients ((0.86±0.38)%, Fig. 3D, 3E, 3F) but lower than that of NC group (CR vs NC, p<0.001; OB vs NC, p = 0.020). The Treg cells percentages of CR group and OB group were of no statistical difference (p = 0.086). There was no statistic difference among the 3 groups (CR (1.89±0.88)%, NR (3.75±1.12)%, OB (1.86±0.67)%, p = 0.103; Fig. 3G, 3H, 3I) when Th17 cells percentages were compared. While all patient groups had higher Th17 cell percentage compared to NC group (p values were 0.038, 0.007, and 0.032 for CR, NR, and OB group respectively). Treg/Th17 cells ratio of CR patients was higher than that of NR patients (CR (1.50±0.48), NR (0.23±0.09), p = 0.025; Fig. 3J, 3K), but no statistic difference was found when compared to OB group ((2.13±0.43), p = 0.086; Fig. 3L). The Treg/Th17 cells ratio of CR group or OB group was lower than that of NC group (CR vs NC, p<0.001; OB vs NC, p = 0.002).

The Treg cells percentage and Th17 cells percentage of OB group stayed stable during the 12 months follow-up (p = 0.949, p = 0.964, respectively), as well as the Treg/Th17 ratio (p = 0.917).

### Patients’ Treg/Th17 Cells at Diagnosis Correlated with their Outcome

**Figure 4:**
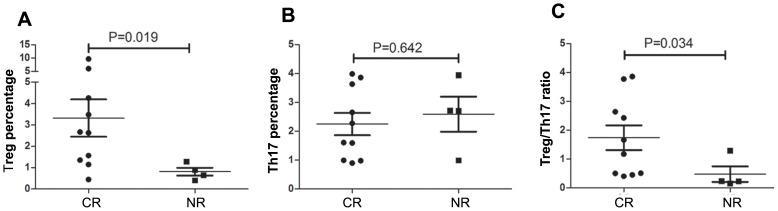
Comparison between CR and NR patients at diagnosis. A. Comparison of Treg cells percentage between CR and NR patients at diagnosis. B. Comparison of Th17 cells percentage between CR and NR patients at diagnosis. C. Comparison of Treg/Th17 ratio between CR and NR patients at diagnosis. *p* value was shown in the figure. CR: complete response; NR: no response.

RT patients were divided into 2 groups according to their subsequent response to corticosteroids. Those who obtained CR had more Treg cells ((3.32±2.77)% vs (0.81±0.37)%, p = 0.019; Fig. 4A) and fewer Th17 cells ((2.24±1.23)% vs (2.59±1.21)%, p = 0.642; Fig. 4B) than those who obtained NR at enrollment, which led to a higher Treg/Th17 ratio in the patients who obtained CR at diagnosis ((1.74±1.36) vs (0.48±0.55), p = 0.034; Fig. 4C). Table 4 showed that there is no statistic difference on sex, age and platelet count difference between the CR and the NR patients.

**Table 4:** Comparisons of multiple parameters at diagnosis between CR and NR patients.

	CR	NR	p value
sex(M:F)	02∶08	03∶01	0.062
age(median,y)	39	55	0.479
platelet count(median,×10^9^/L)	18	10.7	0.67
Treg(mean ± SD,%)	3.32±2.77	0.81±0.37	0.019
Th17(mean ± SD,%)	2.24±1.23	2.59±1.21	0.642
Treg/Th17 ratio (mean ± SD)	1.74±1.36	0.48±0.55	0.034

CR: complete response; NR: no response.

## Discussion

ITP is an immune-mediated heterogeneous disorder. Increasing evidence has shown that T cell-mediated effects play a role in the mechanisms of thrombocytopenia in ITP [1]–[3]. The current study longitudinally followed up the relationship between the ratio of Treg/Th17 and the platelet number. The results showed that Treg/Th17 balance changed toward Th17 in ITP patients. It suggested that the skewing of Treg/Th17 might play a role in pathogenesis of ITP. This finding was similar to the Treg/Th17 imbalance found in juvenile idiopathic arthritis (JIA), systemic lupus erythematosus (SLE) [24], [25], [27]. Higher Th17 cells percentage and Treg cells depletion were linked to more severe JIA disease [24], [25] or SLE flare [27].

Treg cell, as the important regulator of the immune system, has been proven to take part in the pathogenesis of ITP. Liu *et al* [7] compared Treg cells percentage and their suppressive activity between healthy donors and ITP patients, they found that decreased number and function of Treg cells might take part in the immune dysfunction in ITP. In the current study, Treg cells percentage was significantly higher in the normal control group than that in ITP groups (the requiring treatment group and the observation group), which was in accordance with other studies. In addition, our findings showed that the Treg cells percentage of the observation group was significantly higher than that in the requiring treatment group because of the different severities of the disease. Among the treated patients, CR group had more Treg cells than NR patients did, from enrollment to 12 months post treatment, in concert with the clinical difference. Higher percentage of Treg cells correlated with higher platelet count and better clinical outcome. Since we were not able to follow up healthy subjects for 12 months, we compared the Treg cell percentages of CR patients with normal control group at the enrollment. The results showed that Treg cells percentage recovered to the level of observation group at the end of 12 months follow-up, but still statistically lower than that of the normal control. These findings suggested that when ITP occurred, the patients could be naturally divided into favorable and poor prognostic groups based on the Treg cells percentage.

Th17 cells percentage in ITP groups was significantly higher than that in normal control group. One study showed that IL-17A producing CD4^+^ by FCM increased in non-treated ITP patients [14], while another report confirmed increased Th17 cells in ITP patients (both newly diagnosed and CR patients) [16]. The Th17 predominance in ITP disclosed in these studies, which coincide with our results, suggested that Th17 cells contributed to the pathogenesis of the disease. In the current study, the Th17 cells percentages were further followed up in ITP patients of different disease activity in long term. Although the Th17 cells percentages deviated numerically along with the disease activity, there was no statistical difference among ITP groups (CR, NR, and observation) throughout the 12 months period. The comparison between normal control volunteers with CR, NR, or observation group at 12 months post enrollment suggested that Th17 cells increased as long as the disease emerged, which was unrelated to the disease activity, implying an association between Th17 cell elevation and the development of ITP.

It was demonstrated that Treg cells and Th17 cells had a reciprocal relationship. Recent reports have shown that Treg/Th17 ratio may be a useful marker for assessing the severity of diseases in animal models and human diseases, and important mechanisms were postulated to explain the skewed Treg/Th17 ratio [24]–[26]. However, no study has described the significance of the skewed Treg/Th17 ratio by longitudinally following up the patients with ITP, or compared the Treg/Th17 ratio between requiring treatment ITP patients and need no treatment ITP patients. The current study showed that Treg/Th17 balance skewed toward Th17 cells when disease onset. Furthermore, the balance changed along with the disease state, as CR patients’ Treg/Th17 ratio elevated higher than the NR patients’ after treatment. The Treg/Th17 ratio of CR patients kept higher than that of NR patients throughout the 12 months follow-up.

As the Treg/Th17 ratio reflected both the regulatory condition and inflammation status of immune system, we argue that this parameter could be a better indicator of the disease severity. At enrollment, the observation group, as the mild and stable disease state of ITP, had the intermediate Treg/Th17 ratio, which was higher than that of the treated group (severe state) but lower than that of normal control group. During the 12 months follow-up, the platelet count as well as the intermediate Treg/Th17 ratio remained stable in the observation patients. Similarly, CR patients had higher Treg/Th17 ratio at diagnosis when compared with NR patients. The results also showed that the Treg/Th17 ratio of CR patients recovered to the intermediate level, which was the same to the observation patients, but still statistically lower than that of normal control volunteers. The long-term follow-up results of platelet count and Treg/Th17 ratio confirmed that higher Treg/Th17 ratio correlated with higher platelet count and better clinical outcome.

It remains unclear on the definite trigger of ITP, however, the altered pro-inflammation cytokines, especially IL-1β, IL-23, IL-21 and IL-6, may account for the Treg/Th17 imbalance in ITP patients, as they play important role in the differentiation pathway of the two cells [20]. Based on the current knowledge of the interaction between antigen presenting cell (APC) and T cell, we propose that the abnormal APC may be related with the altered Treg/Th17 balance by secreting pro-inflammation cytokines. Furthermore, the involvement of Treg/Th17 change might have effect on the induction of platelet autoantibodies. To investigate these hypotheses, study is undergoing in our institution to assess the relationship of APC with the altered Treg/Th17 ratio, and the production of antibodies by B cells in response to Treg/Th17 cells.

### Conclusions

In summary, our study indicated that the Treg/Th17 ratio might be relevant to the clinical diversity of ITP patients, and this ratio might have prognostic role in ITP patients. We believe that identifying the prognostic role of Treg/Th17 balance, for example, setting up a scored prognostic system based on the Treg/Th17 ratio, is critical for the development of improved clinical management and therapeutic options. Clearly larger clinical trial is needed to be carried out to institute such a prognostic grading system. Despite of the current findings, further work is needed to explore the underlying mechanisms.
